# Cerebrospinal fluid and the early brain development of autism

**DOI:** 10.1186/s11689-018-9256-7

**Published:** 2018-12-13

**Authors:** Mark D. Shen

**Affiliations:** Carolina Institute for Developmental Disabilities, Department of Psychiatry, and the UNC Intellectual and Developmental Disabilities Research Center, University of North Carolina at Chapel Hill School of Medicine, Campus Box 3367, Chapel Hill, NC 27599-3367 USA

**Keywords:** Autism spectrum disorder, Biomarkers, Brain development, Brain enlargement, Cerebrospinal fluid, Early risk signs, Extra-axial cerebrospinal fluid, Glymphatic system, Heterogeneity, Infancy, Lateral ventricles, Neural meningeal lymphatic system, Neuroinflammation, Stratification biomarker

## Abstract

**Background:**

There is currently a renaissance of interest in the many functions of cerebrospinal fluid (CSF). Altered flow of CSF, for example, has been shown to impair the clearance of pathogenic inflammatory proteins involved in neurodegenerative diseases, such as amyloid-β. In addition, the role of CSF in the newly discovered lymphatic system of the brain has become a prominently researched area in clinical neuroscience, as CSF serves as a conduit between the central nervous system and immune system.

**Main body:**

This article will review the importance of CSF in regulating normal brain development and function, from the prenatal period throughout the lifespan, and highlight recent research that CSF abnormalities in autism spectrum disorder (ASD) are present in infancy, are detectable by conventional structural MRI, and could serve as an early indicator of altered neurodevelopment.

**Conclusion:**

The identification of early CSF abnormalities in children with ASD, along with emerging knowledge of the underlying pathogenic mechanisms, has the potential to serve as early stratification biomarkers that separate children with ASD into biological subtypes that share a common pathophysiology. Such subtypes could help parse the phenotypic heterogeneity of ASD and map on to targeted, biologically based treatments.

## Introduction

Until recently, it was thought that the main purpose of cerebrospinal fluid (CSF) was merely to provide protective cushioning of the brain, but new discoveries in the last 5 years have revealed that CSF plays a critical role in brain development and function, both prenatally and throughout the lifespan. It is now recognized that the two primary functions of normal CSF circulation are as follows: (1) delivery of growth factors and other signaling molecules necessary for healthy neural growth [1–4] and (2) cleaning of the brain by removing neurotoxins and metabolic waste byproducts of neuronal function [5–7]. Here, we will briefly review each of these CSF functions and then focus on CSF abnormalities reported in autism spectrum disorder (ASD) and their implications on brain development.

## CSF production and delivery of growth factors

The CSF system originates in the first few weeks of gestation when the neural tube closes and is filled with CSF [8]. As the neural tube elongates, it forms the central canal of the central nervous system (CNS)—with the most rostral end becoming the walls of the lateral ventricles and the caudal end becoming the spinal cord [8]. CSF is continually produced by the choroid plexus in the ventricles, where it delivers signaling molecules to progenitor cells that originate on the apical surface of the ventricles [1–4]. With signaling from growth factors supplied by circulating CSF, these progenitor cells proliferate on the ventricular surface into immature neurons [1–4], which then migrate from the ventricular surface to different layers and regions of the developing cerebral cortex, where finally they will aggregate and differentiate to form identifiable parts of the brain [8]. Thus, the CSF system—and the growth factors that CSF that deliver while circulating through the CNS—play a driving force in regulating early brain development and neural cell proliferation and migration [3].

## CSF absorption and cleaning of neuroinflammation

**Fig. 1:**
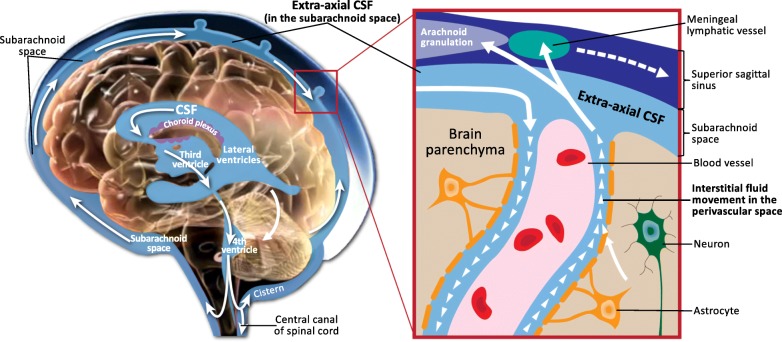
Schematic of CSF circulation, CSF outflow systems, and the anatomy of various CSF compartments. CSF is produced by the choroid plexus in the ventricles, where it delivers growth factors to progenitor cells that originate on the surface of the ventricles, and then proliferate into neurons and migrate to form the cerebral cortex. CSF circulates from the lateral, third and fourth ventricles to the cisterns of the brain, and then flows into the subarachnoid space, where it envelops the cortical convexities of the brain (EA-CSF). Inset box: From the subarachnoid space, there is retrograde influx of CSF into the parenchyma, where CSF and interstitial fluid interact in the perivascular space, alongside blood vessels that course throughout the brain. Astrocytes lining the perivascular space aid in transporting fluid that removes inflammatory waste proteins (e.g., Aβ), which are continually secreted by neurons as byproducts of neuronal activity and would otherwise build up in the brain. Finally, fluid carrying these inflammatory waste products returns to the subarachnoid space (EA-CSF) and drains into meningeal lymphatic vessels and arachnoid granulations.

Throughout early development and the lifespan, the production of CSF needs to be continuously balanced by the corresponding absorption of CSF. The brain continuously produces CSF at a rate of 500 cm^3^ each day [9] and then must be efficiently absorbed and recycled, as the brain typically only holds 150 cm^3^ of CSF at any given time [9]. In fact, a fresh batch of CSF is produced and recycled four times per day [9]. Freshly produced CSF circulates from the lateral, third, and fourth ventricles to the cisterns of the brain and then flows into the subarachnoid space, where it envelops the cortical convexities of the brain. Seventy-five percent of the CSF volume in the brain is located in the subarachnoid space; and 25% is located in the ventricles [9]. From the subarachnoid space, there is influx of CSF into the interstitial space, where CSF and interstitial (ISF) fluid interact within the parenchyma to remove catabolic waste proteins that have been produced as byproducts of neuronal function [5]. Finally, the subarachnoid CSF drains into neural lymphatic vessels [10] and through one-way valves called arachnoid granulations [11]. Thus, while the production of freshly circulating CSF allows for the delivery of nutrients and peptides to neurons, the corresponding absorption of CSF provides the necessary removal of toxic, catabolic waste byproducts of neuronal function [5]. Conversely, perturbations in CSF circulation can impair clearance of harmful substances that accumulate in the brain and lead to neuroinflammation [6]. This “cleaning system” of the brain—and the critical role of normal CSF circulation in this cleaning system—has only recently been discovered. As discussed later in this review, these discoveries have led to new hypotheses about what happens when the CSF system is not functioning normally and the potential consequences of impaired clearance of harmful neuroinflammation [5–7]. See Fig. 1 for a schematic depicting the circulation of CSF, the CSF outflow systems, and the anatomy of various CSF compartments.

## MRI markers of CSF

In vivo structural magnetic resonance imaging (MRI) can measure the volume of different CSF compartments, which can serve as indirect markers of altered CSF production and absorption.

### Lateral ventricle volume in ASD

Deviation from typical levels of CSF production, represented through an enlargement or reduction in lateral ventricle (LV) volumes, has been a research focus in multiple neurodevelopmental disorders [9]. However, the findings related to LV volume in ASD have been inconsistent. Several studies have reported no differences in LV volume in school-aged children [12] and adults with ASD [13, 14], compared to controls. In contrast to these findings of normal CSF volume in the ventricles, there is evidence for increased volume of CSF located outside the ventricles (i.e., CSF volume contained in all of the cisterns and entire subarachnoid space) [14], as well as increased volume of global CSF across the whole brain [15].

### Extra-axial CSF volume in ASD

**Fig. 2:**
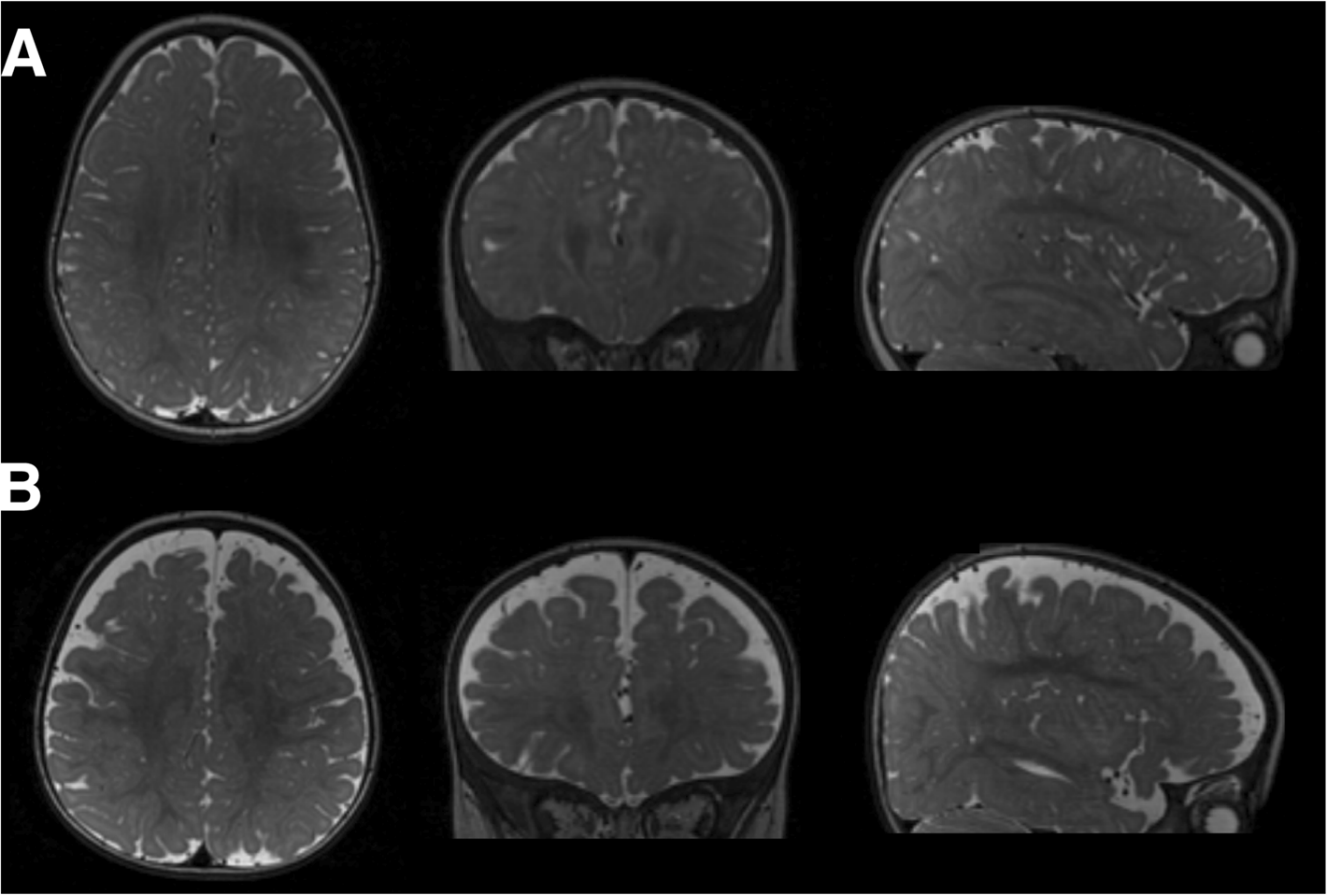
**a** T2-weighted images of an infant with a normal MRI at 6 months of age, who was confirmed as having typical development at 2 years of age. **b** Similar T2-weighted images of an infant with excessive extra-axial CSF at 6 months, who was diagnosed with ASD at 2 years of age. [CSF is indicated as brighter regions in these images. Images are of a horizontal section (left), coronal section (middle), and sagittal section (right) through the brain.]

Studies of infants at high familial risk for ASD have indicated that the defining diagnostic features of ASD, such as social deficits, are not present at 6 months of age, but begin to emerge between 12 and 24 months [16–18]. We recently identified a brain anomaly at 6 months of age, prior to the onset of diagnostic symptoms, in high-risk infants who were ultimately diagnosed with ASD (HR-ASD) [19]. At 6 months, infants who later developed ASD (*n* = 10) had increased “extra-axial CSF,” which is an excessive amount of CSF in the subarachnoid space surrounding the cortical surface of the brain (see Fig. 2). (This study developed a novel method in infant MRIs to quantify the volume of extra-axial CSF (EA-CSF) in the dorsal subarachnoid space above the horizontal plane of the anterior-posterior commissure, thereby avoiding ventral regions that contain cisterns, sinuses, and vasculature that should not be classified as extra-axial CSF.) Increased volume of EA-CSF at 6 months of age preceded the onset of diagnostic symptoms in infants later diagnosed with ASD and remained abnormally elevated at 12 and 24 months of age [19]. Increased EA-CSF was predictive of later autism diagnosis, and greater EA-CSF at 6 months was associated with more severe autism symptoms at the time of diagnosis at 36 months of age [19], suggesting that the severity of this early CSF anomaly is associated with the severity of later autism symptoms.

**Fig. 3:**
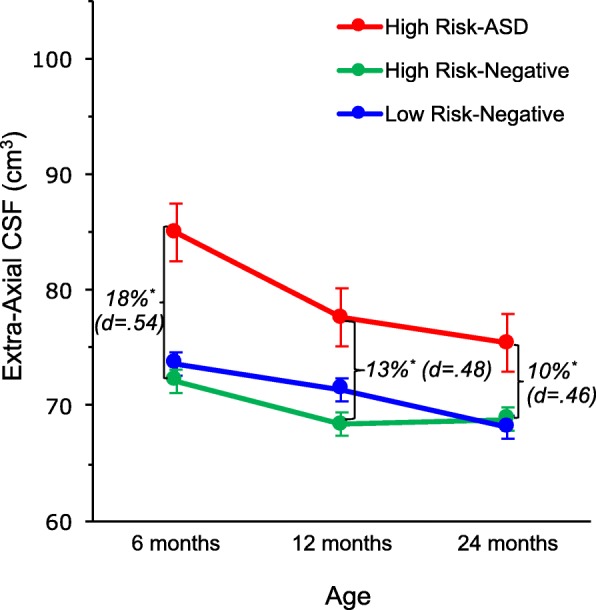
Infants later diagnosed with autism spectrum disorder (ASD) had abnormally increased extra-axial CSF by 6 months, which remained significantly elevated through 24 months. [Least squares means are adjusted for age, sex, total cerebral volume, and scan site. Error bars ± 1 SEM. *p = .005 vs. both control groups (high risk-negative and vs. low risk-negative). Percent differences and Cohen’s d effect sizes are calculated in relation to the high risk-negative group.] (Adapted from Shen et al. [20])

The finding in Shen et al. 2013 [19] was the first MRI report of a structural brain alteration in infants who developed ASD, but it was a relatively small sample (*N* = 55 total infants studied, 10 of whom developed ASD), and thus warranted replication in a larger, independent sample. In Shen et al. 2017 [20], the findings were replicated and extended in a larger, independent cohort of infants (*N* = 343 infants, 47 of whom developed ASD). In this second study, infants who later developed ASD had 18% more EA-CSF at 6 months than the control groups (HR-negative and LR groups). EA-CSF volume remained persistently elevated through 24 months of age, relative to controls (Fig. 3). This replication study included one of the largest longitudinal MRI samples of infants who developed ASD and thus had the opportunity to examine subgroups within ASD to determine if EA-CSF at 6 months could stratify children by the severity of symptoms the children would exhibit at the age of diagnosis. The severity categories were based on well-validated, empirically derived cutoffs on the Autism Diagnostic Observation Schedule (ADOS) that index the severity of autism symptoms [21]. Infants who were later diagnosed with the most severe autism symptoms had a more pronounced increase of EA-CSF—nearly 25% greater EA-CSF at 6 months than controls [20]. Consistent with the first study, the second study demonstrated that the amount of EA-CSF at 6 months of age preceded the onset of autistic symptoms, was predictive of later autism diagnosis, and was associated with severity of later autism symptoms [20].

We recently published a third study of a large cohort of preschool-aged children with ASD (*N* = 159), who were scanned at 2–4 years of age and had 15% more EA-CSF at this age than children with typical development (*N* = 77) [22]. This third cohort of children with ASD extended the findings from the previous infant studies because it comprised both high-risk and low-risk children at preschool-age: (a) children diagnosed with ASD who were “high-risk” like the children in the infant studies (i.e., came from a multiplex family with more than one child with ASD in the family) and (b) children with ASD who were “low-risk” (i.e., came from a simplex family in which they were the only child with ASD). High-risk and low-risk children with ASD had nearly identical volumes of EA-CSF, and both had significantly greater EA-CSF than typically developing controls at 2-4 years of age [22]. In summary, increased EA-CSF appears to be a reliable brain anomaly that has been found in three independent cohorts of children with ASD, regardless of familial risk background, from infancy through preschool age [19, 20, 22].

While these studies were the first to report an association between increased EA-CSF and ASD, several previous reports in the general pediatrics literature have reported an association between increased EA-CSF and motor delays [23–26]. Since early motor delays have also been widely reported in infants who are later diagnosed with ASD [18, 27, 28], the Shen et al. 2017 [20] study tested the hypothesis that EA-CSF would be associated with early motor deficits in ASD. Indeed, using both direct examination and parent-interviews of motor ability, increased EA-CSF at 6 months was significantly associated with deficits in motor ability at 6 months (but not non-motor abilities) in infants later diagnosed with ASD [20]. This is notable given the emerging evidence that motor problems are an early inherent feature of ASD. There are strong genetic associations with motor problems in ASD [29], and coupled with the presence of deficits in fine and gross motor skills at 6 months of age [27, 30] and increased motor stereotypies at 12 months of age [31], this collective evidence points to abnormal development of motor systems prior to the onset of the hallmark diagnostic symptoms. The specificity of the association between increased EA-CSF volume at 6 months and poorer motor skills at 6 months [20] suggests that increased EA-CSF may be related to motor development during the prodromal period in ASD, before behaviors diagnostic of ASD typically arise. Future studies are needed to elucidate the potential relationship between EA-CSF, motor function, and other putative motor systems of the brain (e.g., basal ganglia, cerebellum).

### Relationship between extra-axial CSF and lateral ventricle volume in ASD

In both studies of infants who developed ASD, LV volume was not significantly enlarged compared to controls, despite increased volume of extra-axial CSF [19, 20]. Furthermore, LV volume and EA-CSF volume were not significantly correlated with each other [20]. This is consistent with reports in the general pediatrics literature that increased EA-CSF volume is usually found in the absence of LV enlargement [23, 32, 33], and intracranial pressure is usually normal [11, 23]. LV volume was also found to be highly variable within the ASD group and across infancy, whereas EA-CSF was persistently elevated from 6 to 24 months in infants later diagnosed with ASD [19, 20]. Collectively, the pattern of neuroanatomical findings in infancy is consistent with studies in older children and adults with ASD reviewed above, demonstrating that CSF volume is relatively normal inside the ventricles but abnormally increased in the extra-axial space surrounding the brain. Combined with the finding that lateral ventricle volume and extra-axial CSF volume are not correlated, the extant evidence in ASD indicates there is a decoupling between the volume of CSF in the lateral ventricles and the volume of CSF in the extra-axial space. This pattern of neuroanatomical anomalies raises the possibility that there is an imbalance between CSF production and absorption in ASD, such that normal-sized lateral ventricles may reflect relatively normal CSF production, whereas increased extra-axial CSF may be a potential marker of impaired CSF circulation and absorption. Elucidating the underlying physiology of these anatomical CSF findings will require future studies using animal models or non-invasive CSF flow studies in children.

### Relationship between extra-axial CSF and brain tissue volume in ASD

Is increased EA-CSF volume simply the result of brain tissue loss? In neurodegenerative conditions like dementia, increased extra-axial CSF arises because CSF fills in the subarachnoid space that was previously occupied by atrophic brain tissue [34, 35]. However, in all three studies in infants and preschoolers diagnosed with ASD, there was a robust, *positive* association between extra-axial CSF volume and total cerebral volume [19, 20, 22], with 22% shared variance between these two measures [22]. If increased EA-CSF was due to loss of brain tissue then a *negative* association between CSF and brain volume would be expected instead. Thus, increased EA-CSF in young children with ASD is likely related to different mechanisms than what is observed in degeneration of brain tissue.

While EA-CSF volume has been found to be positively associated with overall brain volume [19, 20], there are possible relationships with other changes in gray and white matter that have yet to be explored. For example, abnormal development of cortical gray and white matter have been reported [36–41] in the same cohort of infants with increased EA-CSF [20]. Furthermore, other studies have reported subcortical abnormalities in periventricular regions (e.g., basal ganglia) [42, 43] that might relate to altered CSF anatomy. Future studies are therefore needed to further interrogate the relationships between EA-CSF, lateral ventricles, and cortical and subcortical gray and white matter anatomy.

## CSF abnormalities in the context of early brain overgrowth in ASD

One of the most consistent findings from previous neuroimaging studies in ASD has been that brain size is significantly enlarged in early childhood, so it is important to evaluate CSF abnormalities during infancy in the context of early brain enlargement. The first direct MRI evidence of brain enlargement prior to age 2 years was reported in the same 2013 cohort of 55 infants (10 of whom developed ASD), who were longitudinally imaged between 6 and 24 months [19]. The HR-ASD group had significantly faster growth trajectories of total brain volume, such that by 12–24 months of age the group had larger brain volumes than the controls, on average. This was the first study to prospectively measure longitudinal brain volumes during infancy in ASD [19].

A larger study evaluated the individual trajectories of 15 HR-ASD infants, who had data at all three serial MRI scans at 6, 12, and 24 months of age [41], compared to a large sample of control infants (91 HR infants who did not develop ASD; 42 low-risk infants). In addition to measuring total brain volume, this study also decomposed brain volume into precise anatomical measures of cortical surface area and cortical thickness, which both contribute to overall brain volume but are controlled by distinct genetic mechanisms [44]. HR-ASD infants had increased rate of cortical surface area expansion from 6 to 12 months, followed by an increased growth rate of total brain volume from 12 to 24 months, compared to controls [41].

Taken together, these infant studies show that infants who later develop ASD have elevated levels of EA-CSF at 6 months [19, 20], increased growth rate of the cortical surface between 6 and 12 months [41], and total brain volume overgrowth between 12 and 24 months of age [19, 41]. Thus, brain changes in ASD are present during the prodromal period prior to diagnosis, preceding behavioral differences. At 6 months of age, brain size is normal but there is an excessive amount of EA-CSF [19, 20]. This is a time when the first behavioral differences in ASD are detectable, including motor delays [18, 27, 28], and excessive EA-CSF at 6 months was associated with early motor deficits at 6 months [20]. Between 6 and 12 months of age, there is rapid expansion of cortical surface area [41], which is concurrent with the onset of sensory and attention problems, such as deficits in visual reception [27] and orienting to salient social cues in the environment [45]. Between 12 and 24 months, there is increased growth rate of total brain volume [19, 41], which was reported to be associated with autism-specific social deficits [41]. Thus, early changes in brain development in the first year of life coincide with the age when early sensorimotor and visual orienting differences tend to emerge, which are followed by social deficits in the second year of life and the consolidation of behaviors that are diagnostic of ASD [46].

## The impact of CSF dysfunction on brain development

How might increased extra-axial CSF in infancy and early brain overgrowth be related? CSF circulation serves as a means of transporting important growth factors and signaling molecules throughout the brain that are required for the normal development of the neocortex, such as insulin-like growth factors (IGF1 and IGF2) [1, 3, 4, 9]. Increased EA-CSF volume is a reflection of stagnant or reduced circulation of CSF, as evidenced by consistent findings from several MRI studies that measured the dynamic flow of CSF (using non-invasive diffusion imaging [47] or injected isotopes and contrast agents [19, 23, 24, 48]). Stagnation of CSF leads to the accumulation of neuromodulators in brain tissue that may alter the extracellular environment of neurons and impact their growth and function [5, 49]. An imbalance between CSF production and absorption alters the concentration of these factors and could alter cortical development [50]. For example, stagnation of CSF flow in animal models leads to an alteration of neurogenesis and premature migration of progenitor cells from the apical surface of the ventricles [50]. An imbalance in concentration between IGF1 and IGF2 can result in opposing brain phenotypes of microcephaly and macrocephaly in animal models [3]. Indeed, there is evidence that the composition of CSF drawn from the subarachnoid space in infants with increased extra-axial CSF has a markedly higher protein concentration compared to CSF drawn from the ventricles or spinal column [51], and also compared to CSF in normal infants [48]. Future studies are needed to test the hypothesis that stagnant or elevated EA-CSF in ASD has a different composition of trophic growth factors (IGF1, IGF2) [1, 3, 4, 9].

## The impact of CSF dysfunction on the clearance of neuroinflammation

Is it possible that increased accumulation of CSF over the surface of the brain leads to neuroinflammation? The primary function of continuous CSF outflow is the removal of inflammatory byproducts of brain metabolism, such as amyloid-β (Aβ) and tau protein [6, 9]. In normative brain development, the amount of extra-axial CSF in the subarachnoid space increases from birth to 7 months, declines between 12 to 24 months, and is minimal by 24 months [52]. In ASD, the extant evidence indicates that extra-axial CSF is abnormally elevated until 3 years of age [19, 20, 22], which suggests that the normal mechanisms for outflow of CSF may be aberrant in infants who develop ASD.

There are three clearance systems that are responsible for CSF outflow and clearance of Aβ (see Fig. 1 for a schematic of the various CSF outflow systems). First, in the mature brain, return of subarachnoid EA-CSF to the venous circulation takes place through reabsorption into arachnoid granulations—one-way valves that drain into the dural venous sinuses (e.g., superior sagittal sinus) [53]. However, arachnoid granulations are not open at birth and only mature over the first 18 months of life [54, 55]. The immaturity of arachnoid granulations in infancy may cause CSF to accumulate in the subarachnoid space, leading to elevated extra-axial CSF volume [56, 57]. Second, beyond the arachnoid granulations, there is new evidence that CSF can exit the brain in another way. Two-photon imaging studies from the past few years have indicated that bulk flow of CSF and interstitial fluid (i.e., fluid in the interstitial space) contributes to a larger portion of Aβ clearance than previously thought [5–7, 58]. The flow of fluid—through the interstitial space where it clears extracellular Aβ, and exiting through the subarachnoid space—is facilitated by astroglial aquaporin-4 (AQP4) channels and thus is named the glymphatic (glial + lymphatic) system [5–7, 58]. Third, the recent discovery of the meningeal lymphatic system provides another clearance route for CSF and inflammatory proteins [10]. These meningeal lymphatic vessels provide a direct path between the nervous and immune systems for immune cells to exit the CNS [59] (see Fig. 1). Because these clearance systems act together to drive Aβ from the brain, alterations in any given system could contribute to altered neurophysiology and accumulation of neuroinflammation [59].

### Potential links between CSF dysfunction, the immune system, and brain development in ASD

The link between CSF flow, neuroinflammation, and brain development has been informed by recent discoveries in neurodegenerative disorders. Failure of Aβ clearance is increasingly recognized in the pathogenesis of Alzheimer disease [59]. The pathological hallmark of Alzheimer disease is the accumulation of toxic proteins—Aβ plaques and tau tangles [59]. Now, there is emerging evidence that CSF clearance of Aβ is impaired in both early-onset and late-onset forms Alzheimer disease, resulting in excessive accumulation of toxic forms of Aβ [59]. In fact, Aβ deposition can be elevated during the presymptomatic period of Alzheimer disease, years or even decades before the presentation of the hallmark neurological and cognitive symptoms [59, 60].

On the other end of the lifespan, there is a marked increase in CSF production in the first year of normal brain development [61], which may not be a problem for typical infants whose CSF production is balanced by proper absorption through the mechanisms discussed above. However, there may be an imbalance between CSF production and CSF drainage in infants with excessive extra-axial CSF who later develop autism [19, 20]. CSF is recycled at a much slower rate in early life [9], and there is a higher ratio of CSF-to-brain volume in infancy [61]. Collectively, these factors contribute to the infant brain having less ability than the mature brain to eliminate inflammatory metabolites and toxins, making it more vulnerable to damage if the CSF system is perturbed [9].

In ASD, there are three lines of converging evidence that support a potential link between increased extra-axial CSF, impaired CSF circulation, and buildup of neuroinflammation (e.g., Aβ). First, there is mounting evidence of increased levels of Aβ in individuals with ASD, which has been found in neurons from postmortem brain tissue, blood, and peripheral CSF [62–65]. Second, sleep problems are commonly found in ASD [66, 67], and disrupted sleep hinders the flow of CSF and its ability to clear Aβ [6]. Consistent with this proposed link between sleep problems and CSF abnormalities, we found that worse sleep problems in preschool children with ASD (*N*=159) were associated with greater EA-CSF volume [22]. During natural sleep, there is a 60% increase in the influx of CSF compared to the awake state, as the exchange of CSF between the interstitial space and the subarachnoid space is accelerated during sleep [6]. This increased flow of CSF during normal sleep facilitates the increased clearance of Aβ, which is continually secreted by neurons and needs to be continually removed by the efficient flow of CSF [6]. Thus, it is possible that sleep disturbances in ASD may impair the normal restorative function of sleep to clear inflammatory byproducts that accumulate in the awake brain. Of course, this proposed mechanism needs to be tested with animal models in order to elucidate the pathophysiology underlying this reported relationship between sleep problems and increased EA-CSF in ASD [22].

Third, the recent discovery of the meningeal lymphatic system [10], and its reliance on normal drainage of CSF, raises the possibility that the lymphatic system and immune system are involved in increased extra-axial CSF. A recent report [68] demonstrated that impaired function of meningeal lymphatic vessels resulted in (a) reduced drainage of CSF from the subarachnoid space into the meningeal lymphatics (with no change in ventricular volume), (b) decreased circulation of fluid through the parenchyma and clearance of macromolecules, (c) accelerated Aβ accumulation in the parenchyma and subarachnoid space, and (d) cognitive deficits in learning and memory. These results suggest that normal drainage of CSF by the meningeal lymphatics is necessary for proper cognitive function. The meningeal lymphatic vessels may serve as a direct path for immune cells to exit the CNS, and dysfunction of these vessels might have important implications for neurological conditions associated with altered immune responses [59]. This may be relevant for neurodevelopmental disorders like ASD, given the growing evidence for the interplay between the immune system and central nervous system in neurodevelopmental disorders [69] and that immune dysfunction is commonly found in individuals with ASD [70–74].

## Conclusions and future directions

Currently, diagnosis of autism spectrum disorder (ASD) relies on the presence of behavioral impairments that do not emerge until the latter part of the first and during the second year of life [17], and thus, diagnosis is not typically made until 3–4 years of age [75]. Early behavioral differences between those who develop ASD and those who do not have only been identified at the group-average level (e.g., see [27]), and early behavioral markers are not sensitive nor specific enough for individual-level prediction of later ASD diagnosis [76]. Consequently, it is common practice to not initiate treatment until after behavioral diagnosis of ASD. However, there is emerging consensus in ASD that earlier intervention is more effective than later intervention [77–80].

Thus, there is a need for both early and biologically derived markers for ASD in infancy to help identify which children need what type of treatment. It is important to identify not only *prediction* biomarkers, but also *stratification* biomarkers to parse the phenotypic heterogeneity in ASD, which is a well-recognized impediment to developing targeted treatments. For example, a fully cross-validated machine learning algorithm relying on the amount of EA-CSF volume at 6 months predicted later ASD diagnosis at 24 months with 66% sensitivity and 68% specificity [20]. This prediction algorithm was then externally validated in a separate sample of infants [19] (in order to test the algorithm on an independent dataset), which yielded 80% sensitivity and 67% specificity in predicting ASD diagnosis on the basis of EA-CSF volume at 6 months [20]. This identical prediction algorithm using EA-CSF was recently validated in a third independent sample of preschool-aged children with 83% positive predictive value (PPV), 84% sensitivity, and 65% specificity [22]. The findings of these studies emphasized the importance of moving beyond group-level differences toward individual-level prediction, which indicated that CSF abnormalities are present at 6 months of age, prior to the onset of the defining behavioral features of ASD. Given the heterogeneity of ASD, it is unlikely that increased EA-CSF is present in all children with ASD, as the sensitivity and specificity metrics were not high enough for EA-CSF to serve as a *single stand-alone marker* for all cases on the autism spectrum. However, the replication and reliability of the findings between three independent cohorts (consisting of both high- and low-risk children with ASD) [19, 20, 22] indicate that increased EA-CSF at 6 months could be a potential early stratification biomarker that delineates one biological subtype of ASD that shares a common underlying biology.

In order to validate EA-CSF as a potential stratification biomarker, several future studies must be conducted to (a) determine its specificity (by comparing to other neurodevelopmental disorders), (b) elucidate the underlying biology (using animal models and identifying genetic associations), and (c) test potential mechanisms using experimental approaches such as those established by studies described above of the glymphatic and meningeal lymphatics systems [5, 6, 10, 68]. For instance, identifying CSF flow abnormalities in genetically defined syndromes of ASD—and then conducting mechanistic experiments in the animal models of such syndromes (e.g., Fragile X, Tuberous Sclerosis, Dup15q, Angelman syndrome)—would help the field take the next step toward teasing apart biology and developing targeted treatments.

The phenotypic heterogeneity in ASD has hampered efforts toward targeted treatments, which has led to major initiatives by the child psychiatry field and the U.S. National Institute of Mental Health to identify biological subtypes of ASD [81]. Thus, there is a need for stratification biomarkers that can separate children into ASD subtypes that share a common pathophysiology. The clinical utility of such stratification biomarkers would be to parse the autism spectrum into clinically significant subtypes that map on to specific, mechanistically targeted treatments [82].

## Abbreviations

ADOS: Autism Diagnostic Observation Schedule
AQP4: Astroglial aquaporin-4
ASD: Autism spectrum disorder
Aβ: Amyloid-β
CNS: Central nervous system
CSF: Cerebrospinal fluid
EA-CSF: Extra-axial cerebrospinal fluid
HR: High-risk for autism by virtue of having an older sibling with autism
HR-ASD: High-risk infant who was subsequently diagnosed with ASD (HR-ASD)
IGF: Insulin-like growth factor
LR: Low-risk for autism by virtue of having no first- or second-degree relatives with autism or psychiatric disorders
LV: Lateral ventricle
MRI: Magnetic resonance imaging
PPV: Positive predictive value
